# Evaluation of cytotoxicity and antibacterial activity of different pulp capping liners

**DOI:** 10.1080/26415275.2023.2287019

**Published:** 2023-12-27

**Authors:** Zohre Moradi, Mahdi Abbasi, Abbas Bahador, Mir Saeed Yekaninejad, Xaniar Mohammadi Khanghah, Amirahmad Pahlavan Hoseini, Ladan Ranjbar Omrani

**Affiliations:** a Dental Research Center, Dentistry Institue, Restorative Dentistry Department, School of Dentistry, Tehran University of Medical Sciences, Tehran, Iran; b Oral Microbiology Laboratory, Department of Microbiology, School of Medicine, Tehran University of Medical Sciences, Tehran, Iran; c Department of Epidemiology and Biostatistics, School of Public Health, Tehran University of Medical Sciences, Tehran, Iran; d Department of Maxillofacial Surgery, Faculty of Dentistry, Tabriz University of Medical Sciences, Tabriz, Iran; e Department of Prosthodontics, Islamic Azad University Dental Branch of Medical sciences, Dental School, Tehran, Iran

**Keywords:** Dental pulp capping, MTT formazan, ACTIVA BioACTIVE-BASE LINER, anti-bacterial agents, *Streptococcus mutans*, *Lactobacillus casei*

## Abstract

This study compares the cytotoxicity and antibacterial activity of five pulp capping liners. This *in vitro* study was conducted on Fuji II LC glass ionomer, Dycal, Calcimol LC, TheraCal LC, and ACTIVA BioACTIVE. For cytotoxicity, the (MTT) methyl thiazolyl tetrazolium assay was performed on 3 samples from each group of human dental pulp cells (HDPSCs) after 24 h of incubation. The direct contact test (DCT) for antibacterial activity, 6 samples (3 for each material, and 3 for negative control), from each liner were made to evaluate *Streptococcus mutans* (*S. mutans*), *Lactobacillus casei* (*L. casei*), and *Lactobacillus acidophilus* (*L. acidophilus*). Data were analyzed by one-way ANOVA and Tukey’s post-hoc test (alpha = 0.05). Data analysis showed that the cytotoxicity of the materials was significantly different (*p* < 0.001). Fuji II LC and ACTIVA BioACTIVE showed strong cytotoxicity, TheraCal LC moderate cytotoxicity, and Dycal and Calcimol LC slight cytotoxicity. The analysis also revealed a significant difference among the materials regarding antibacterial activity (*p* < 0.001). Tukey’s test showed that the mean percentage of reduction in colony count was significant for all liners compared with the positive control (*p* < 0.001). The mean percentage of reduction in colony count for Dycal was significantly greater than that of Fuji II LC (*p* = 0.014), Calcimol LC (*p* = 0.003), and TheraCal LC (*p* = 0.001). ACTIVA BioACTIVE did not significantly differ from the other materials as regards antibacterial activity. Dycal showed significantly higher antibacterial activity than the other materials.

## Introduction

Pulp vitality is imperative to preserve the integrity of the tooth structure and ensure its normal physiological function. Thus, there is an increasing demand for methods to preserve pulp vitality in the treatment of deep carious lesions and even after pulp exposure [1].

Direct or indirect capping of a vital pulp is a commonly used dental procedure to seal the areas close to the pulp chamber, induce reparative dentin formation, and preserve pulp vitality [2]. A number of factors affect the success of pulp capping treatment such as the antibacterial activity of the capping liner, bleeding control, absence of cytotoxicity of the pulp capping liners, and their biocompatibility [2].

Biocompatibility of direct pulp capping liners is among their most important properties because they remain in contact with the pulp tissue for a long period of time [3,4].

The bacteria present in deep carious lesions can induce severe inflammatory reactions in the dental pulp and even lead to pulp necrosis [5]. Thus, prevention of bacterial infection is an important step to improve the efficacy of pulp capping treatment for deep carious lesions. By prevention of bacterial contamination of the dental pulp, the pulp tissue can use its innate capacity for regeneration and repair [6].

Another concern with respect to cariogenic bacteria is their leakage through the restoration-cavity wall interface. Several materials have been suggested to seal the pulp chamber in deep cavities and in cases with pulp exposure [7]. Calcium hydroxide paste (aqueous suspension) was the first material suggested for direct pulp capping. It was the standard capping liner to preserve pulp vitality for a long period of time. At present, different formulations of calcium hydroxide and mineral trioxide aggregate are most commonly used for pulp capping [8,9].

Dycal is a radiopaque, hard, self-setting calcium hydroxide mixture that does not interfere with composite polymerization. Calcium hydroxide has a high pH which is responsible for its bactericidal activity and can neutralize the acidic pH of carious lesions [10,11].

Glass ionomer (GI) is another liner, which has an optimal biocompatibility if it is not in direct contact with the pulp tissue [12]. GI releases fluoride and has a low primary pH [12]. It creates a good cavity seal, and prevents the leakage of cariogenic bacteria and tooth demineralization [13].

ACTIVA BioACTIVE liner has an ionized, bioactive resinous hydrophilic matrix, modified with polyacrylic acid [14]. It also possesses the optimal physical and esthetic properties of composite resins, and can release greater amounts of calcium, phosphorus, and fluoride compared with conventional and resin-modified GI. According to the manufacturer, ACTIVA BioACTIVE induces the formation of mineral apatite crystals at the tooth-restoration interface, and this natural remineralization process fills the micron-scale gaps at the tooth-restoration interface, prevents secondary caries, and seals the restoration-tooth margin against microleakage. It responds to pH alterations in the oral cavity by releasing phosphorous ions in acidic conditions to alkalize the environment and enhance tooth remineralization [15].

Calcimol LC is a light-cure, radiopaque, resin-modified material with a hydrophobic matrix and a calcium hydroxide base for pulp capping. It can release calcium and enhance the formation of secondary dentin and hydroxyapatite. Due to high pH [7–9], it has antibacterial properties as well and can enhance healing [16]. Moreover, it can serve as a protective acid-resistant pulp capping liner in the process of acid etching [17].

TheraCal LC is a light-cure, radiopaque and moisture-resistant resin-modified pulp capping liner with a calcium silicate base [18]. It has a more hydrophilic matrix compared with Calcimol LC, which results in greater calcium release. Also, it induces the formation of secondary dentin bridges and hydroxyapatite, and improves the seal as such. Its alkaline pH enhances the formation of apatite and protects the dental pulp [18].

Sahin et al. [19] compared the efficacy of three pulp capping liners for indirect pulp capping of primary teeth. After exfoliation of the teeth, histological sections were made and assessed. They found that TheraCal LC had a significantly lower success rate than Biodentine and Dycal considering the impaired integrity of the odontoblastic layer and the degree of pulpitis. Kim et al. [20] compared the biocompatibility of ProRoot MTA, Biodentine, TheraCal LC, and Dycal for human dental pulp stem cells by the methyl thiazolyl tetrazolium (MTT) assay. They indicated that ProRoot MTA had the highest biocompatibility followed by Biodentine, TheraCal LC, and Dycal. Poggio et al. [16] assessed the antimicrobial activity of several pulp capping liner by the agar disc diffusion method. They showed that the antibacterial activity against *Streptococcus mutans (S. mutans*) was higher in MTA-Angelus compared with TheraCal LC and Dycal; while, the lowest antibacterial activity was observed for Biodentine, Calcimol LC, and Calcicur.

Although cavity liners help to restore pulp vitality, adding resinous material may modify the products released by these materials which can affect the surrounding tissues as they pass through the dentin canals to the pulp during and after the polymerization process. As there is lack of studies comparing the antibacterial activity and biocompatibility of liners with different basic structures modified by resinous materials and existing controversy in the literature on this topic, this study aimed to compare the cytotoxicity and antibacterial activity of novel bioactive pulp capping liners in comparison with other conventional cavity liners using the direct contact method against *S. mutans, L. casei* and *L. acidophilus*. These bacteria, in the form of polymicrobial suspension, are often used for assessment of the antimicrobial efficacy of restorative materials *in vitro* because they are responsible for development of secondary caries, and their interactions can bring about different effects.

## Materials and methods

### Study design and groups

For assessment of antimicrobial activity, the tests were repeated in triplicate to minimize measurement errors. Three tubes were allocated to each pulp capping liner, and three tubes to the negative control group (a total of 30 tubes for 5 groups) plus three tubes for the positive control group, yielding a total of 33 tubes. For each liner 6 samples were prepared.

### Preparation of specimens for the MTT assay and antibacterial testing

The materials were applied in a mold measuring 4 × 1 mm between two transparent matrix bands.

Dycal (Dentsply Caulk Milford, DE, USA): The base and catalyst paste of Dycal were mixed in equal ratios and allowed to set at 26 °C for 3.5 min according to the manufacturer’s instruction.

Fuji II LC (GC, Hasunuma-Cho, Itabashi-Ku, Tokyo, Japan): Fugi II LC GI was mixed according to manufacturer’s instruction and light-cured for 20 s using an LED curing unit with a light intensity of 1200 mW/cm^2^ (Woodpecker, Guilian, Guangxi, China).

ACTIVA BioACTIVE base/liner (Pulpdent, Watertown, MA, USA): The base and catalyst were mixed by the tip of the auto-mix syringe, injected directly into the mold, and cured for 20 s.

TheraCal LC (Bisco Inc., Schaumburg, IL, USA): The material was directly injected into the mold and cured for 20 s.

Calcimol LC (Voco GmbH, Cuxhaven, Germany): The material was directly injected into the mold and cured for 20 s.

The specimens were then removed from the mold, placed in the tubes using a spatula and gamma-sterilized (25 kGy). The 1:1 concentration of extracted materials were evaluated for cytotoxicity and antibacterial activity of liners.

### Antimicrobial testing

#### Preparation of bacterial suspension

A mixed suspension of *S. mutans* (ATCC 35668), *L. casei* (ATCC 334), and *L. acidophilus* (ATCC 4356) was used in this study. The microorganisms were obtained from the microbial bank of Pasteur Institute of Iran in lyophilized form, and remained refrigerated at 4 °C. To prepare the microbial suspension, *L. acidophilus* and *L. casei* were cultured on MRS agar, and *S. mutans* was cultured on mitis salivarius agar. The plates were incubated under anaerobic conditions in a Gas-Pak at 37 °C for 48 h. Pure colonies were used to prepare a bacterial suspension with 0.5 McFarland standard concentration containing 1.5 x 10^8^ colony forming units (CFUs)/mL and confirmed by spectrophotometry (optical density [OD] 600: 0.08–0.1) [21]. The standard suspension was then diluted to create a suspension with the desired concentration for the direct contact test. To validate the culture method, a mixed planktonic suspenson containing *S. mutans, L. casei,* and *L. acidophilus* in equal proportions was checked on selective media [22].

#### Exposure of bacterial suspension to the capping liners

To assess the antimicrobial activity of the capping liners by the direct contact method (DCT), the specimens were placed in 1.5 ml sterile Eppendorf tubes containing microbial suspension at 5 x 10^5^ CFUs/mL concentration and then the tubes were incubated at 37 °C for 24 h. Empty tubes containing microbial suspension alone served as the positive control, and tubes containing the capping liners in culture medium without the microorganisms served as the negative control (for assessment of possible microbial contamination). After anaerobic incubation, bacterial proliferation was evaluated by counting the colonies in the test and control groups by serial dilution and culture. Each test was performed in triplicate. The tubes had a glass fiber membrane with 0.45-µm pore size allowing only the passage of liquids and not bacteria and material particles. Thus, it had no confounding effect on the outcome variable (colony count).

### Cytotoxicity testing

#### Preparation of extracts

The samples were prepared as mentioned above in a mold with 5 mm diameter and 3 mm height and were sterilized with ultraviolet (UV) light for 30 min to prevent contamination. All the prepared samples for the MTT assay were incubated at 37 °C for 24 h immediately after mixing. According to ISO 10993-12 standards for preparing the release of fluids from solid form materials, the discs were immersed into 15 ml of Dulbecco’s modified Eagle medium (DMEM) (Vitrocell Embriolife, Campinas, SP, Brazil) and incubated for 24 h at 37 °C in a humidified 5% CO_2_ environment. The extract material solutions were passed twice through the 0.22 μm filter to remove small particles, and the material extracts were obtained for the cytotoxicity tests according to ISO 19993-5 [23].

### Cytotoxicity assessment by the MTT assay

The proliferation rate of DPSCs growing in the presence of the different liner extracts was evaluated using the MTT assay. Human dental pulp stem cells (HDPSC) were obtained from the cell bank of Iranian Genetic Resources. Frosted cells were in a nitrogen tank, and they were transferred to a 37 °C water bath and defrosted. Subsequently, culture medium was added to the cells, they were centrifuged, and the previous medium was extracted. The cell suspension was transferred to a flask containing DMEM (Gibco), supplemented with 15% fetal bovine (FBS; Gibco) and 1% penicillin/streptomycin (Gibco), and they were then incubated at 37 °C with 5% CO_2_ and 95% humidity. The third passage of the cells, after reaching 70–80% confluence in the flask, were gradually separated from the bottom of the dish using 0.25% trypsin. 100 µL of the cell suspension containing 10,000 cells was added to each well of a 96-well plate. They were then starved for 24 h with serum free medium in an incubator at 37 °C in 5% CO_2_ and 100% humidity. The serum-free medium was replaced with the liner extracts. Three wells containing 10Ùª volume of dimethyl sulfoxide (DMSO) served as the positive control [24]. Three other wells containing cells and culture medium served as the negative control.

The MTT assay was performed to assess the cytotoxicity of the materials after 24 h. After incubation, extracts were removed, 10 µL of the MTT dye solution was added to each well, and the well-plate was incubated for 4 h under the aforementioned conditions. The MTT medium was replaced by DMSO (100 µL) and glycerin buffer (20 µL) to dissolve the insoluble purple formazan crystals. To assess cell proliferation, the plates were shaken for 10 min, and the optical density of the wells (experimental wells as well as positive and negative controls) was measured by a spectrophotometer (Biotek, Epoch, USA) at 570 nm wavelength.

The cell viability was compared with the control, and the result was defined as non-cytotoxic (>90% cell viability); slightly cytotoxic (60–90%); moderately cytotoxic (30–59%); and strongly cytotoxic (<30%) [25].

### Statistical analysis

The effect of type of material on cytotoxicity and colony count (CFUs/mL) was analyzed by one-way ANOVA followed by the Tukey’s HSD test (for pairwise comparisons) using SPSS version 25 at 0.05 level of significance.

## Results

### Antimicrobial activity

The negative control group (capping liners in culture medium broth without microorganisms) showed no bacterial growth, indicating no contamination during the experimental process and accurate sterilization. The positive control group (bacteria without the capping liners) showed bacterial proliferation. Table 1.

**Table 1.** Mean and standard deviation (SD) of the colony count after antibacterial testing.

Material	Mean (SD)	Min	Max
Calcimol LC	14.93 (0.83)	14.00	15.60
Dycal	10.86(1.30)	9.50	12.10
TheraCal LC	15.46(1.23)	14.10	16.50
ACTIVA BioACTIVE	13.06(0.15)	12.90	13.20
Fuji II LC	14.16(0.98)	14.00	15.60
Control	24.2 (0.95)	23.20	25.10

One-way ANOVA found a significant difference in the antibacterial activity of the groups.

The maximum mean reduction in colony count was noted for Dycal followed by ACTIVA BioACTIVE, while the minimum percentage of reduction in bacterial count was noted for TheraCal. Also, the mean percentage of bacterial reduction for Dycal was significantly greater than for Fuji II LC (*p* = 0.014), Calcimol LC (*p* = 0.003), and TheraCal LC (*p* = 0.001). However, no significant difference was found between Dycal and ACTIVA BioACTIVE (*p* = 0.139).

No significant differences were found between Fuji II LC, Calcimol LC, and TheraCal LC (*p* > 0.05), and ACTIVA BioACTIVE did not differ significantly from any other material (*p* > 0.05).

### Cytotoxicity and biocompatibility

**Figure 1.**
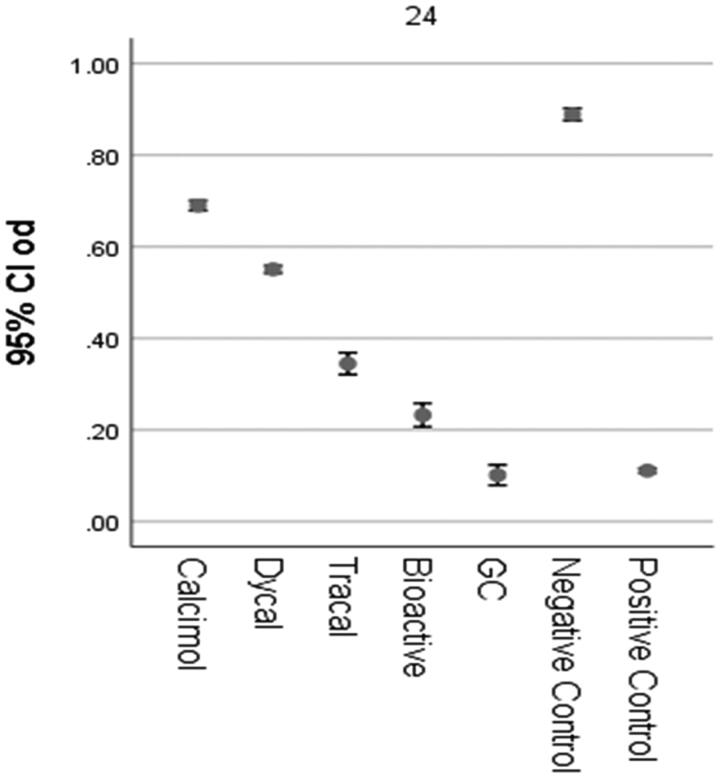
The viability of cells in the study groups by the MTT assay after 24 hours.

Figure 1 shows the viability of cells in the study groups. One-way ANOVA showed a significant difference in biocompatibility among the groups.

Considering the normal distribution of data, pairwise comparisons were performed by the Tukey’s test. The statistical analyses showed that the cytotoxicity of the capping liners in the first 24 h was as follows (from the highest to the lowest): Fuji II LC > ACTIVA BioACTIVE > TheraCal LC > Dycal > Calcimol LC. Dycal and Calcimol LC were slightly cytotoxic, while TheraCal LC was moderately cytotoxic, whilw Fuji II LC and ACTIVA BioACTIVE were strongly cytotoxic.

## Discussion

By the advances in dental material science and marketing technology in dentistry, a number of novel pulp capping liners are annually introduced to the market. Thus, assessment of antibacterial activity and cytotoxicity of these novel products is a priority.

Pulp capping is a primary dental procedure for preservation of pulp vitality. Bacteria left in the cavity may lead to secondary caries or pulpal injury after restoration. *S. mutans, L. casei*, and *L. acidophilus* are responsible for development of secondary caries [26]. Additionally, Pulp capping liners are in direct or indirect contact with the pulp tissue. The materials released from the pulp capping liners and their constituents can play a fundamental role in resolution or progression of pulpitis. Thus, biological properties and cytotoxicity of biomaterials are highly important for their clinical application.

Considering the polymicrobial nature of caries and synergy between the microorganisms, a microbial suspension consisting of three more resistant microorganisms was used in this study since the interaction of several bacteria in the clinical setting yields different results [21].

Conventionally, several methods are used for assessment of antibacterial properties of dental materials such as the agar diffusion and direct contact tests [26]. The agar diffusion test is semi-quantitative and not highly sensitive [26]. The size of growth inhibition zones depends on the ability of the capping liners to diffuse in agar, and thus, this test can be used only for water soluble materials [26]. The direct contact method introduced by Weiss et al. [27] is a quantitative and reproducible method for simulation of the contact of microorganisms with dental materials [28]. Its main advantage compared with the agar diffusion test is better control of the confounding factors. Also, it can be used for insoluble materials [28]. This study used the direct contact test to assess immediate antimicrobial activity of the cavity limers after contact with the microorganisms [21].

This study compared the antibacterial activity of resin modified bioactive pulp capping liners with conventional cavity liners. The results showed that all materials had antibacterial activity compared with the control group, which was in line with other studies. [29–31]. Dycal, a conventional cavity liner, showed significantly higher antibacterial activity than Fuji II LC, Calcimol LC, and TheraCal LC. The mechanism of antibacterial activity of Dycal is based on the release of free hydroxyl radicals, pH rise, and reaction with biological molecules [32]. The cell membrane enzymes of microorganisms are inactivated at pH values over 9, and thus, their biological activity decreases [32].

Fuji II LC is a biocompatible liner (if not in direct contact with the pulp tissue) with acid-base setting reactions. Its antibacterial effects are due to the release of fluoride and low primary pH, preventing bacterial proliferation and metabolism [15]. Fluoride directly prevents bacterial proliferation by deactivating enzymes involved in cellular metabolism [15].

Calcimol LC is a resin-modified pulp capping liner with a hydrophobic matrix and a calcium hydroxide base. It has antibacterial effects due to high pH. Its high pH increases the release of hydroxyl ions and changes the integrity of the cell membrane by chemically degrading the organic components, phospholipids, and unsaturated fatty acids [17]. Bacteria such as streptococci and staphylococci can only proliferate at a pH between 3 to 8. However, the pH of calcium hydroxide liners such as Calcimol LC is over 10, exerting antibacterial effects [17]. According to Ericson and McComb, light-cure calcium hydroxide-based materials have lower antibacterial activity than self-cure compounds, which may be due to the light-curable nature of constituents and absence of self-cure chemical agents, resulting in generation of lower amounts of free radicals and ions, which is in agreement with the present findings [33].

TheraCal LC is a resin-modified calcium silicate-based cement. It has a more hydrophilic matrix compared with Calcimol LC, which results in greater release of calcium hydroxide and a greater rise in pH [18], exerting antibacterial effects. The present results confirmed this statement. However, Poggio et al. [16] demonstrated that TheraCal LC had lower antibacterial effects on *Streptococcus salivarius* and *Streptococcus sanguinis* than Dycal, but its effect on *S. mutans* was similar to the effect of Dycal. However, this difference was not significant in the study by Yalcin et al. [29]. Also, Poggio et al. [16] indicated comparable antimicrobial efficacy of TheraCal LC and Calcimol LC, which is in accordance with the present findings. Arias-Moliz et al. [34] explained the reason for this similarity to be the high pH of both materials. According to Petrolo et al. [35] TheraCal showed superior antibacterial effects compared with Fuji Ii LC although the difference did not reach statistical significance. This variation in the results may be due to different methodologies such as incubation in anaerobic conditions, which causes the generation of smaller amounts of free radicals. Arias-Moliz et al. [34] discussed that the antibacterial activity of TheraCal is influenced by its pH.

In 2013, ACTIVA BioACTIVE was introduced to the market. The manufacturer claims that it has antibacterial activity due to the presence of acidic monomers [36]. However, the present results did not confirm this claim. Although it showed a significant antibacterial activity compared with the positive control group, the difference between ACTIVA BioACTIVE and the other liners was not significant in this respect, which may be due to the methodology of the study such as the concentration of the liners and prevention of the antibacterial activity on the surface of the liners by the presence of the glass fiber membrane, which did not allow direct contact of the liners with the bacteria. Thus, further studies are warranted on this topic [16]. Since a number of clinical factors affect the antimicrobial activity of materials in the clinical setting, long-term studies are required to evaluate the antibacterial properties of pulp capping liners against the main microorganisms responsible for secondary caries and pulpitis. Moreover, the pH of ACTIVA BioACTIVE should be compared with other pulp capping liners in presence of dentin.

Cell-based studies are often performed to find out whether a novel dental material has significant positive effects on cell proliferation or has cytotoxic effects and leads to cell death [20]. Cell-based studies are also extensively performed to assess the receptor binding and signalling pathway events, which may include gene expression, transfer of cell components, or supervision of function of organelles. In this study, MTT assay was used. The mechanism of action of the MTT assay is based on the conversion of yellow MTT salt to purple formazan crystals in viable cells, that are quantified by a spectrophotometer [37]. Human dental pulp stem cells (HDPSCs) were used in this study. Since they are highly sensitive, low cytotoxicity of materials for HDPSCs indicates their acceptable biocompatibility [38].

The present results indicated that after 24 h, Calcimol LC had the lowest cytotoxicity among the tested materials, a result which differs from the findings of Poggio et al. [16,26] in 2014 and 2015, respectively. Poggio et al. [16,26] evaluated the odontoblasts of rats while the present study was conducted on HDPSCs. Use of different cell types and higher sensitivity of HDPSCs compared with odontoblasts may explain the difference in the results. Since Calcimol LC should be light-cured, its cytotoxicity due to its high pH is lower than that of Dycal, and calls for further investigations.

According to the present results, the cytotoxicity of Dycal was higher than that of Calcimol LC and lower than that of the other materials. This result is also in contrast to the results of Poggio et al. [26]. They explained that the antibacterial activity of Dycal depends on the release of hydroxyl ions. Since the hydroxyl ions attack both the microorganisms and host cells, they adversely affect the mitochondrial activity of the host cells and exert cytotoxic effects. Poggio et al. [26] used MDPC odontoblast-like cells. Use of a different cell type can explain the variations in the results. In another study, Poggio et al. [26] reported higher cytotoxicity of Dycal compared with TheraCal LC. They used rat MDPC odontoblasts and Alamar Blue cytotoxicity assay, which may be the reason for different results obtained in their study compared with the present investigation. The high pH of Dycal and calcium hydroxide-based materials causes a necrotic layer in direct contact with the pulp, which may be responsible for cytotoxicity of Dycal.

According to the present results, TheraCal LC had higher cytotoxicity than Calcimol LC and Dycal, and lower cytotoxicity than ACTIVA BioACTIVE and Fuji II LC. These findings are in contrast to the results of Kim et al. [20]. They found that cells in contact with Dycal showed lower viability than those in contact with TheraCal LC. The present results, however, were in line with those of Adiguzel et al. [39] and Zakerzade et al. [4] who reported higher cytotoxicity of TheraCal LC compared with other calcium silicate-based cements. They attributed this finding to the presence of unpolymerized compounds, resinous structure of TheraCal LC, low Portland Cement content in the composition of TheraCal LC, and insufficient wetting of calcium silicate power. Furthermore, due to the completion of hydration process in TheraCal LC, release of calcium hydroxide does not occur in sufficiently high amounts.

ACTIVA BioACTIVE showed higher cytotoxicity than Calcimol LC, Dycal, and TheraCal LC but its cytotoxicity was lower than that of Fuji II LC. It appears that bioactive particles may have a role in reduction of cytotoxicity of resin-modified GI. The current results did not agree with the findings of Jun et al. [40]. They concluded that ACTIVA BioACTIVE, which is a light-cure liner with glass particles, had a cytotoxicity comparable or higher than that of Dycal. The majority of light-cure materials containing bio­active liner are polymerized with the help of photo-initiators such as camphorquinone. When these components penetrate into the tissue, they may exert toxic effects on the adjacent cells. Considering the constituents of bioactive liners and the mechanism of polymerization of unpolymerized monomers, silica, fluoride ions, and camphorquinone may be responsible for induction of cytotoxicity.

The present results showed that Fuji II LC had the highest cytotoxicity after 24 h of all the materials tested, which is in agreement with the findings of other studies [41,42]. Low pH of Fuji II LC compared with other materials may explain its high cytotoxicity. Also, ions released from it (F-, Ca2+, Al3+, Si4+, Sr2+) can be toxic in high concentrations; however, above all, organic materials such as resin monomers released from GI are believed to be mainly responsible for its high cytotoxicity.

While all materials in this study had antibacterial effects, it seems that Calcimol LC is as safe a material as Dycal for pulp preservation in direct and indirect pulp capping procedure. TheraCal LC had moderate cytotoxicity and must therefore be used with caution, while ACTIVA BioACTIVE had a cytotoxicity comparable to that of Fuji II LC, and ACTIVA should not be used near the pulp and also needs protective liners in these situations.

One of the limitations of this study is the use of a culture-based method to evaluate the antibacterial activity, especially in polymicrobial models. Other complementary assays to biofilm assessment methods are suggested for future studies.

## Conclusion

Within the limitations of this study, the following conclusions can be drawn:

While all the liners tested had antibacterial effects it seems that Calcimol LC is as a safe material as Dycal for direct pulp capping while Fuji II LC and ACTIVA BioACTIVE were strongly cytotoxic and should not be used in direct contact with pulp.
